# Coping Styles, Mental Health, and the COVID-19 Quarantine: A Nationwide Survey in Poland

**DOI:** 10.3389/fpsyt.2021.625355

**Published:** 2021-03-11

**Authors:** Agata Kołodziejczyk, Błazej Misiak, Dorota Szcześniak, Julian Maciaszek, Marta Ciułkowicz, Dorota Łuc, Tomasz Wieczorek, Karolina Fila-Witecka, Sylwia Chladzinska-Kiejna, Joanna Rymaszewska

**Affiliations:** ^1^Division of Psychotherapy and Psychosomatic Medicine, Wroclaw Medical University, Wroclaw, Poland; ^2^Department of Genetics, Wroclaw Medical University, Wroclaw, Poland; ^3^Department of Psychiatry, Wroclaw Medical University, Wroclaw, Poland; ^4^Practice of Family Doctors M.V. Domanscy, Wroclaw, Poland

**Keywords:** coping styles, quarantine, mental health, COVID-19, stress

## Abstract

**Background:** The outbreak of the novel coronavirus COVID-19 that began from March 2020 is yet to be contained. Consequences of the ongoing pandemic may have a negative impact on the mental health of affected individuals. This particularly refers to those quarantined. Since the COVID-19 pandemic is currently one of the biggest health issues worldwide, a higher demand emerges for research concentrating on the worsening of psychological well-being among the general and the quarantined population, as well as on individual coping strategies that may moderate the occurrence of psychopathologies.

**Method:** Data were collected within the first weeks of the COVID-19 pandemic in Poland. Participants represented quarantine (+) and quarantine (–) groups. Quarantine (+) group, different from quarantine (–), consisted of people who experienced it themselves or someone close to them did after contacting an infected individual. To measure psychopathological symptoms a General Health Questionnaire (GHQ-28) was used. For measuring PTSD symptoms, the Impact of Event Scale-Revised (IES-R) was used. This study followed the coping strategies manifested among the participants using the MiniCope questionnaire.

**Results:** A total of 2,036 individuals participated in this study. Quarantine (+) individuals had significantly higher total and subscales GHQ-28 scores (anxiety, insomnia, and somatic symptoms) as well as a higher IES-R arousal score. The quarantine (+) individuals were more likely to use self-distraction as a coping strategy. This research identified positive and negative correlations between presented coping styles and manifested psychopathology.

**Conclusion:** This nationwide study suggests occurrence of negative effects on mental health due to the COVID-19 pandemic and quarantine. It is observed on most of the measured psychopathological symptoms. The present research provides a line of action that should be followed in the future in case of another epidemic and in the event restrictions like quarantine have to be introduced again.

## Introduction

During the last months of 2019, worldwide attention has focused on an outbreak of COVID-19. In the following weeks, the virus began to spread, causing the World Health Organization to declare the pandemic state on March 11, 2020 (1). Till this day, this epidemiological crisis remains one of the most pressuring health issues worldwide.

The COVID-19 outbreak raised a number of questions and concerns, as did the other pandemics that occurred before. Taking into account human, economic, and social costs and aftermaths of, for example, the SARS epidemic in 2003 (2) or the Ebola outbreak in 2009 (3), researchers are doing their best to prevent not only the spread of the disease but also negative psychological consequences that people experience as a result of being infected themselves or when reacting to worldwide fear and feelings of uncertainty connected with the applied restrictions (4, 5), changes in daily routine, and prolonged stress conditions. Consequently, the psychosocial cost and the impact of the ongoing pandemic on the general population cannot be stressed enough.

In the reports that are covering an ongoing crisis, mental well-being has been indicated as severely affected among the general population (6–9). Reasons for this phenomenon were listed as: grief, loss, unstable economics, uncertainty about the future, stigmatization, and disruption of social support systems. Considering the emotional distress caused by the COVID-19 pandemic, it comes with no surprise that psychopathological symptoms are being detected among people of every affected country and culture (10–12). Most commonly observed themes of psychological responses toward consequences of the COVID-19 outbreak include: anxiety, fears, depression, anger, guilt, grief, loss, and post-traumatic stress reactions (6–8, 13, 14). Despite this, the reasons for these psychological difficulties may differ depending on the extent of restrictions experienced.

Most of the countries affected by the COVID-19 proceeded with all kinds of social restrictions, with quarantine being one of the most isolating and aggravating one (15, 16). Its purpose is to protect the population's physical health, but it often causes the worsening of psychological well-being (17–20). Being quarantined is connected with abrupt changes in daily life, mobility limitations, and disruption of social interactions, contributing to severe stress-related responses, anxiety, depression (7, 8), post-traumatic stress symptoms (PTSS) (14, 21), insomnia, anger, fear of being discriminated or stigmatized (16), low self-esteem, and a lack of self-control (15). Subsequently, available data on this matter denote quarantine as a potential factor for a mental-health deterioration (17).

Furthermore, to understand the emotional and cognitive responses of the general population to the COVID-19 pandemic, as well as the psychopathology it causes, it has become a principal objective to observe and detect individual responses and coping strategies that appear during this stressful time. So far, researchers have observed many stress-related responses among the general population that appeared to be adaptive, as well as maladaptive (19, 20). These responses can have an impact on psychological well-being, contributing to unfavorable mental-health outcomes. Moreover, coping strategies that general population adopt during an ongoing pandemic seems to vary depending on applied restrictions and these restriction differ depending on the level of the threat of being infected (19, 22). Additionally, it has become important to make a distinction between quarantine and isolation. Although quarantine is often referred to when talking about general isolation recommendations, in fact the quarantine concerns people who had a direct contact with an infected person and do not yet know whether they were infected or not, whereas isolation is recommended to already infected individuals^1^. Subsequently, even though researchers have already addressed problems evoked by the recommendations concerning quarantine (16, 19, 22), the results may differ when taking into account its exact definition and consequences of uncertainty about one's health. And since the SARS-CoV-2 virus outbreak remains at the center of attention worldwide, it seems crucial to examine different coping responses that have been adopted by the general population and their role in developing psychopathological symptoms. This should be researched especially among those quarantined due to an actual infection or to a real threat of manifesting symptoms within days after contacting someone infected.

This international interest in COVID-19 research should be followed simultaneously globally and regionally. Since mental, behavioral, and cognitive responses to the pandemic may differ depending on cultural and geosocial factors, nationwide studies on this issue are needed.

Consequently, the purpose of this study was to measure psychological outcomes of the COVID-19 pandemic, as well as coping strategies that people tended to engage in during the outbreak. Most importantly, it was designed to examine potential differences in those features between people who were quarantined and those who were not.

## Materials and Methods

### Participants

Data were collected using an online survey which was launched on March 16, 2020 and lasted until April 26, 2020. The study was initiated 12 days after the first case of SARS-CoV-2 was confirmed in Poland. It covered a rapid increase in COVID-19 cases as well as social restrictions, which followed it, including social isolation and quarantine. Quarantine was defined as a protective measure that restricts the movement of people who were in contact with COVID-19 positive individuals and therefore await whether or not they got infected. Participants over 18 years old, from all administrative parts of Poland were invited to fill in the survey, which was shared throughout social media and email addresses. Participants represented quarantined as well as not quarantined groups. Data analyses considered completed surveys only. The study was approved by the Ethics Committee at the Wroclaw Medical University in Poland and was performed in accordance with the Declaration of Helsinki. All participants provided written informed consent.

### Measures

The survey consisted of the sociodemographic section, the author's questionnaire, and three standardized questionnaires: the MiniCope Questionnaire (MiniCope), the Impact of Event Scale-Revised (IES-R) and the General Health Questionnaire (GHQ-28). The sociodemographic questionnaire followed data on general demographic characteristics, such as age, sex, place of residence, marital status, education, and profession. The author's questionnaire included questions related to exposure to COVID-19, the availability of protective measures, quarantine, change of working hours, and place of employment during the pandemic, as well as feelings associated with it. For the purpose of this study, it also consisted particularly important questions concerning history of being quarantine. Participants were asked whether they experienced quarantine or someone close to them did, meaning they themselves or their relatives were in fact in contact with someone infected and as a result experienced uncertainty and anxiety about their health. Ultimately, this information became the dividing criterion for researched population making it possible to compare differences between them in relation to psychopathological symptoms and coping strategies.

The MiniCope (23) is a 28-item questionnaire used to measure coping strategies. Its main purpose is to assess typical ways of responding and feeling in situations of severe stress among the general population. It consists of 14 subscales that record the following coping strategies: active coping (items: 2 and 7), planning (items: 14 and 25), positive reframing (items: 12 and 17), acceptance (items: 20 and 24), sense of humor (items: 18 and 28), turning to religion (items: 22 and 27), seeking emotional support (items: 5 and 15), seeking instrumental support (items: 10 and 23), self-distraction (items: 1 and 19), denial (items: 3 and 8), venting (items: 9 and 21), substance use (items: 4 and 11), behavioral disengagement (items: 6 and 16), and self-blame (items: 13 and 26). Answers to each item are based on a four-point Likert scale (0-I hardly ever do that, 1-I rarely do that, 2-I often do that, 3-I almost always do that). Scores of each subscale range from 0 to 3. Higher scores indicate higher tendency to use specific coping strategies while being under severe stress.

The IES-R (24) is a 22-item questionnaire used to identify post-traumatic stress-related reactions. These are divided into three subscales, representing groups of symptoms related to post-traumatic stress disorder (PTSD): intrusion (items: 1, 2, 3, 6, 9, 14, 16, 20), hyperarousal (items: 4, 10, 2, 15, 18, 19, 21), and avoidance (items: 5, 7, 8, 11, 13, 17, 22). Answers to each item are given using five-point Likert scale (0-not at all, 1-in small extent, 2-restrainedly, 3-mostly, 4-definitely yes). A general score above 30 indicates the occurrence of PTSD symptoms as a result of coping with a traumatic event.

The GHQ-28 (25) is a 28-item questionnaire developed to record minor psychiatric disorders in the general population. It consists of four subscales: somatic symptoms (items 1, 3, 4, 8, 12, 14, 16), anxiety and insomnia (items 2, 7, 9, 13, 15, 17, 18), social dysfunction (items 5, 10, 11, 25, 26, 27, 28), and severe depression (items 6, 19, 20, 21, 22, 23, 24). The GHQ-28 items are rated using a four-point Likert scale (0-not at all, 1-no more than usual, 2-rather more than usual, 3-much more than usual). The total score ranges between 0 and 84. Higher scores indicate higher levels of distress. Scores above 24 are typically interpreted as a sign of significant psychopathology and are labeled as positive GHQ-28 in this paper (25).

### Data analysis

Before data analysis, participants were divided into two groups. The first group [quarantine (+) individuals] included participants surveyed during the COVID-19 quarantine (*n* = 41, 2.0%) and those who had reported having a relative or close friend undergoing quarantine due to the COVID-19 quarantine (*n* = 383, 18.8%). These participants were clustered together due to underrepresentation of participants that had been quarantined during the survey. In turn, the second group [quarantine (–) individuals] included participants who reported a negative history related to their own quarantine (meaning they did not have contact with an infected individual and as a result did not experience uncertainty about their potential health status) and a lack of relatives of close friends referred to quarantine (*n* = 1,612, 79.2%) (for the above-mentioned reasons). Both groups were compared using the Mann-Whitney U test (continuous variables) and the chi-square of Fisher exact tests (categorical variables). In order to test correlations between IES-R scores, GHQ-28 scores, and the MiniCope scores, Spearman's rank correlation coefficients were used. The analysis of co-variance (ANCOVA) was performed to investigate the differences in the level of psychopathological symptoms (GHQ-28 and IES-R scores) between quarantine (+) and quarantine (–) individuals, after co-varying for potential confounding factors. The significance level for all results was set at 0.05. All analyses were performed using *R*^2^ (version 3.5.3) with MASS^3^ and MatchIt^4^ packages.

## Results

### Participants

General characteristics of the participants, with differences between quarantined and not quarantined groups, are presented in Table 1. The quarantine (–) group was more likely (at the trend level significance) to report being a primary caregiver of somebody with special needs. No other significant differences, with respect to demographic data between groups, were observed.

**Table 1:** General characteristics, psychopathological symptoms and coping strategies among quarantine (+) and quarantine (–) groups.

	**Quarantine(+)** ***n*= 424**	**Quarantine(–)** **n= 1612**	***P*-value**
Age	38.88 ± 12.37	39.93 ± 12.75	0.172
Sex			0.066
Women	344 (81.1%)	1238 (76.8%)	
Men	80 (18.9%)	374 (23.2%)	
Being in a relationship			0.635
Yes	327 (77.1%)	1223 (75.9%)	
No	97 (22.9%)	389 (24.1%)	
Having children			0.234
Yes	215 (50.7%%)	875 (54.1%)	
No	209 (49.3%)	740 (45.9%)	
**Taking care of a disabled person**			**0.050**
Yes	80 (18.9%)	239 (14.9%)	
No	343 (81.1%)	1389 (85.1%)	
**GHQ**			
**Total score**	30.2 ± 15.11	27.68 ± 14.86	**<0.001**
**Positive**	63.4 % (269)	53.5 % (862)	**<0.001**
**Somatic symptoms**	7.5 ± 4.62	7.04 ± 4.57	**<0.001**
**Anxiety and insomnia**	10.16 ± 5.65	9.02 ± 5.4	**<0.001**
Social disfunction	8.49 ± 3.57	8.32 ± 3.48	0.236
Severe depression	3.6 ± 4.02	3.3 ± 3.9	0.097
**IES**			
Total score	1.71 ± 0.81	1.62 ± 0.78	0.056
**Arousal**	1.74 ± 0.92	1.63 ± 0.9	**0.020**
Intrusion	1.75 ± 0.96	1.65 ± 0.94	0.063
Avoidance	1.63 ± 0.77	1.58 ± 0.77	0.289
**MiniCope**			
Active coping	2.1 ± 0.64	2.05 ± 0.64	0.093
Planning	2.12 ± 0.65	2.09 ± 0.63	0.357
Positive reframing	1.75 ± 0.74	1.75 ± 0.71	0.728
Acceptance	2.02 ± 0.64	1.99 ± 0.6	0.143
Sense of humor	1.04 ± 0.64	1 ± 0.59	0.406
Turning to religion	0.96 ± 0.99	0.96 ± 0.97	0.809
Seeking emotional support	1.78 ± 0.77	1.72 ± 0.76	0.098
Seeking instrumental support	1.71 ± 0.74	1.67 ± 0.75	0.219
**Self-distraction**	1.73 ± 0.7	1.65 ± 0.67	**0.029**
Denial	0.74 ± 0.64	0.71 ± 0.63	0.403
Venting	1.54 ± 0.61	1.47 ± 0.61	0.074
Substance use	0.62 ± 0.76	0.56 ± 0.73	0.160
Behavioral disengagement	0.73 ± 0.57	0.73 ± 0.61	0.733
Self-blame	1.27 ± 0.81	1.29 ± 0.82	0.614

*Statistically significant results (p < 0.05) were marked with bold characters. Quarantine (+) - individuals being quarantined or reporting a history of quarantine among relatives and/or friends, quarantine(–) -individuals not being quarantined and not reporting a history of quarantine among relatives and/or friends; IES, Impact of Event Scale; GHQ, general health questionnaire*.

### Psychopathological Symptoms and Coping Strategies Among Quarantined and Not Quarantined Individuals

The results of both quarantined and not quarantined groups showed presence of relevant psychopathology according to GHQ-28 questionnaire (scores above 24)—with a mean of 30.2 and 27.68 respectively (Table 1). Quarantine (+) individuals had significantly higher total GHQ-28 and specific GHQ-28 subscales scores (anxiety and insomnia as well as somatic symptoms) as well as the IES-R arousal score. The frequency of positive GHQ-28 scores was also significantly higher in this group of participants. The quarantine (+) individuals were considerably more likely to use self-distraction as a coping strategy.

The ANCOVA revealed a significant group effect [quarantine (+) vs. not quarantine (–) individuals] on the level of the following psychopathological outcomes: somatic symptoms, anxiety, and insomnia, as well as the GHQ-28 total score, intrusion, hyperarousal, and IES-total score after co-varying for the effects of potential confounding factors (Table 2). It was yet again observed that quarantine (+) group manifested more psychopathological symptoms. The same effect was observed in relation to two manifested coping strategies: active coping and self-distraction. Moreover, a significant independent sex effect in all ANCOVA models was observed, except for the active coping strategy. The age effect was found to be significant in all the ANCOVA models. Lastly, the effect of caring for someone disabled appeared to be meaningful for the GHQ-28 total score and psychopathological symptoms, such as anxiety, insomnia, somatic symptoms, and hyperarousal.

**Table 2:** The ANCOVA models testing for the association between quarantine, psychopathological manifestation and coping styles after co-varying for potential confounding factors.

	**Quarantine (+)**	**Age**	**Sex**	**Caring for a disabled person**
IES – intrusion	*F*= 5.145, ***p*****= 0.023**	*F*= 15.930, ***p*****<0.001**	*F*= 76.616, **p<0.001**	F=0.810, p=0.368
IES – arousal	*F*= 4.499, ***p*****=0.034**	*F*= 28.974, ***p*****<0.001**	*F*= 76.687, **p<0.001**	F= 4.579, **p=0.032**
IES – total score	*F*= 5.599, ***p*****=0.018**	*F*= 13.086, ***p*****<0.001**	*F*= 79.884, **p<0.001**	F= 2.085, p=0.149
GHQ-28 – anxiety and insomnia	*F*= 4.368, ***p*****=0.037**	*F*= 15.030, ***p*****<0.001**	*F*= 107.641, **p<0.001**	F=7.557, **p=0.006**
GHQ-28 – somatic symptoms	*F*= 4.954, ***p*****=0.026**	*F*= 10.769, ***p*****=0.001**	*F*= 103.092, **p<0.001**	F= 9.995, **p=0.002**
GHQ-28 – total score	*F*= 3.724, **p=0.054**	*F*= 28.095, ***p*****<0.001**	*F*= 86.980, **p<0.001**	F= 6.751, **p=0.009**
MiniCOPE – active coping	*F*= 4.714, **p=0.030**	*F* = 10.194, ***p*****=0.001**	*F*= 0.007, p=0.934	F= 0.724, p= 0.395
MiniCOPE – self-distraction	*F*= 4.229, **p=0.040**	*F* = 7.174, ***p*****=0.007**	*F*= 81.022, **p<0.001**	F= 1.052, p=0.305

*Significant effects (p < 0.05) were marked with bold characters. IES, Impact of Event Scale (and its subscales), GHQ, the General Health Questionnaire (and its subscales); Quarantine (+) – participants who have experienced quarantine themselves or someone close to them did*.

### Correlations Between Psychopathology and Coping Strategies

Performed analyses showed several statistically significant correlations between psychopathological symptoms and coping strategies (Table 3). Positive correlations were found between maladaptive coping strategies, such as denial, discharging, substance use, behavioral disengagement, self-blame, and psychopathological symptoms measured by GHQ-28 and IES-R questionnaires. Moreover, a positive correlation was found between the act of turning to religion and IES-R scores. This indicates a relationship between turning to religion and PTSD symptoms among the researched population. We have also observed positive correlation between seeking instrumental support and PTSD symptoms.

**Table 3:** Correlations between psychopathological symptoms and coping strategies in general population.

	**Psychopathological symptoms**							
	**GHQ-somatic symptoms**	**GHQ- anxiety and insomnia**	**GHQ- social disfunction**	**GHQ – severe depression**	**IES- total**	**IES- Intrusion**	**IES- Avoidance**	**IES- Hyperarousal**
**MiniCope**								
Active coping	−0.149^**^	−0.137^**^	−0.214^**^	−0.254^**^	−0.098^**^	−0.081^**^	−0.031	−0.136^**^
Planning	−0.163^**^	−0.150^**^	−0.217^**^	−0.242^**^	−0.112^**^	−0.108^**^	−0.039	−0.147^**^
Positive reframing	−0.244^**^	−0.259^**^	−0.322^**^	−0.327^**^	−0.0174^**^	−0.211^**^	−0.023	−0.228^**^
Acceptance	−0.158^**^	−0.167^**^	−0.227^**^	−0.209^**^	−0.153^**^	−0.166^**^	−0.029	−0.175^**^
Sense of humor	−0.084^**^	−0.107^**^	−0.120^**^	−0.069^**^	−0.069^**^	−0.100^**^	0.038	−0.090^**^
Turning to religion	0.017	0.033	−0.039	−0.026	0.087^**^	0.076^**^	0.099^**^	0.067^**^
Seeking emotional support	−0.053^**^	−0.033	−0.138^**^	−0.128^**^	−0.043	−0.024	−0.039	−0.045^*^
Seeking instrumental support	0.028	0.051^*^	−0.040	−0.026	0.056^*^	0.078^**^	0.011	0.058^**^
Self-distracting	0.124^**^	0.140^**^	0.009	0.088^**^	0.183^**^	0.140^**^	0.232^**^	0.137^**^
Denial	0.257^**^	0.275^**^	0.206^**^	0.273^**^	0.355^**^	0.304^**^	0.232^**^	0.308^**^
Venting	0.220^**^	0.248^**^	0.109^**^	0.193^**^	0.291^**^	0.269^**^	0.227^**^	0.277^**^
Substance use	0.237^**^	0.245^**^	0.166^**^	0.234^**^	0.253^**^	0.248^**^	0.134^**^	0.266^**^
Behavioral disengagement	0.302^**^	0.304^**^	0.331^**^	0.444^**^	0.330^**^	0.319^**^	0.195^**^	0.345^**^
Self-blame	0.273^**^	0.300^**^	0.295^**^	0.438^**^	0.398^**^	0.416^**^	0.224^**^	0.389

Statistically significant results: *p < 0.05 and **p < 0.01. GHQ, the General Health Questionnaire, IES, Impact of Event Scale.

In turn, negative correlations were found between psychopathological symptoms and adaptive coping strategies, such as active coping, planning, acceptance, positive reframing, and sense of humor. Furthermore, we found negative correlations between seeking emotional support and symptoms, such as somatic symptoms, social disfunction, severe depression, and hyperarousal connected with PTSD. No other statistically relevant correlations were observed.

## Discussion

Our research confirmed negative effects of the COVID-19 pandemic as well as ordered quarantine and its consequences on mental health among the Polish population. These were observed with regard to somatic symptoms, anxiety and insomnia, social dysfunction, severe depression, and PTSD symptoms. These findings are in agreement with those from similar studies concentrating on psychopathological consequences of the pandemic that have been performed during previous epidemics and, what is more relevant, during the ongoing one. Indeed, the results of other studies concentrating on the COVID-19 effect on general population's well-being have provided evidence of the following psychological problems: stress load and associated somatic symptoms (26), anxiety and fear (7, 11, 27), PTSD symptoms (14, 21, 27, 28), depression (8, 11, 21, 28) and insomnia (29). Studies concerning previous epidemics have revealed these symptoms as predictors and facilitators of post-pandemic psychiatric disorders, such as panic attacks, psychosis, alcoholism, and even suicide attempts (30, 31). Subsequently, there is a high demand for following such outcomes during the existing pandemic in order to prevent possible future psychiatric morbidity.

Additionally, we were able to recognize coping strategies amid the ongoing stressful event among studied population and their correlations with observed psychopathological symptoms. However, most importantly, we compared participants with respect to a history of quarantine and stress related to it as people who were ordered to quarantine faced a real threat of being infected and, as recent studies show, this threat combined with severe restriction to stay home may contribute to worsened well-being (32). We provided data on significantly worse mental health in people who personally experienced quarantine, or know someone close who did, in comparison to people without such experiences. Differences between these groups particularly referred to somatic symptoms connected to stress, anxiety, insomnia, and PTSD symptoms of intrusion and hyperarousal. The “quarantined group” was much more likely to manifest those psychopathological symptoms.

Such outcomes concerning the quarantined population are supported by other research on similar aspects (33, 34). Hence, even though psychopathological symptoms manifest among the general population during an epidemic, studies show that those symptoms are far more common among quarantined individuals and their families (15, 32, 35, 36). So far, much of the available data concentrated on the effect of quarantine among health-care workers (2, 31). What's important is that these studies have defined quarantine as being isolated from loved ones in order to make sure they didn't put families in danger of being infected and in the same time could continue on working with COVID-19 patients (22). Our study concentrated on quarantine only applying to those who were ordered to quarantine because they have been in a real danger of being infected after contacting a COVID-19 positive person and in result were experiencing a high level of anxiety toward their own and their loved one's safety. That indeed, according to research, may have a negative impact on psychosocial well-being (32). Subsequently, presented results regard not only medical but general population.

Our findings are in agreement with those obtained by Pancani et al. (37), who found deteriorated psychological well-being due to the COVID-19 quarantine among the Italian population. Although, it must be pointed out that in mentioned research, quarantine has been referred to when talking about general isolation restriction, whereas in presented research we focused on individuals ordered to quarantine after contacting an infected person and for that reason direct comparison cannot be performed. Nevertheless, it has been already confirmed that psychological degradation seems to manifest in difficulty to concentrate, boredom, feeling of loneliness (36), insomnia (15, 38), anxiety and specific fears (38), depression (15, 21), lack of self- control (15), and PTSD symptoms (17, 18, 21).

Presented data as well as data obtained from previous pandemics on quarantine effect on mental well-being must be considered and taken into account by the policymakers in order to adopt such restrictions, if needed in the future, without causing any unnecessary psychological harm to affected individuals.

Our results, concerning coping strategies, may find further explanation in the already mentioned paper by Pancani et al. (37). In there, the authors contribute mental-health deterioration among quarantined to limited living space and social contact and inconveniences they provoke. This might have made self-distraction even more troublesome among tested population and could have contributed to psychopathological symptoms occurrence since self-distracting connects with both cognitive and emotional avoidance that often lead to miscommunications and conflicts, which in turn promote negative psychological symptoms (32). Moreover, being quarantined and using self-distraction is often connected with higher exposer to media, which was also denoted as one of the reason for mental health to degrade among general population (7).

On that account, differences regarding coping strategies between two distinguished groups that were observed in the presented study need to be further discussed. It seems that people who experienced quarantine, or know a close relative or a friend who did, are more likely to use self-distraction amid traumatic events. These results are in accordance with other studies. For instance, in their meta-analysis, Chew et al. (6) introduces coping mechanisms most often presented among populations facing pandemics, with self-distraction being the most commonly used avoiding strategy. Many studies show that the latter (39) might lead quarantined individuals to manifest psychopathological symptoms. For instance, the study by Main et al. (40) provided results suggesting that an avoiding style of handling stress contributes to the occurrence of negative mental symptoms. Therefore, it seems crucial to propose solutions thanks to which those quarantined would use less avoidant coping strategies. This might be achieved by introducing psychosocial interventions targeted at those quarantined as well as by providing clear administrative rules for handling quarantine and making it easier to access medical help or information. These recommendations are in agreement with those proposed by Cullen et al. (41).

Yet another relevant outcome of the presented study includes correlations between coping strategies and psychopathological symptoms among the general population. Our research suggests a positive correlation between turning to religion as a coping strategy and PTSD symptoms, which contradicts other studies concentrating on this matter. In his paper, Rabelo et al. (42) proves that religion was one of the biggest facilitators of psychological well-being among the studied population during the Ebola outbreak. In another study (43), information specific to cultural and religious beliefs seemed to have contributed to better mental health among the SARS epidemic survivors. Yet another study (26) indicated that spirituality rather than religiousness was a way of transcending victimhood in order to regain self-empowerment among epidemic survivors. All these results show the impact of religion and spirituality on preserving good mental health during the pandemic. On that account our findings seem unfitted. However, our findings may be associated with the time that our survey was carried out. During that period, restrictions concerning closed churches and limited access to religious events around Poland were introduced. This prevented the Polish population from engaging in any religious event and limited their access to the religious movement's support.

Furthermore, according to our data, seeking instrumental support as a coping strategy had a negative impact on mental health. This may have also been worsened by global miscommunication, contradictory recommendations, and incoherent information about the virus (44), which has been denoted as a globally unsettling issue by the World Health Organization. Negative effects of limited access to essential personal protective equipment and support from health authorities on psychological state, for instance, has been denoted by Delgado (45). This is also pointed out in a study by Jakovljevic (46), who illustrated the huge impact of global mistrust in officials who often mismanaged the COVID-19 outbreak. He also points at media and how they depicted the pandemic, with many misinterpretations and conspiracy theories, which can validate misconception, anxiety, fear, and mass confusion. The present study was conducted during ongoing work on a uniform medical and economic policy in our country. That is why this seems to be the likely reason for Poles' dissatisfaction with the instrumental support they received. This especially refers to those quarantined, who's mental state, as described, presented even worse. This might be potentially connected with their greater dependence on administrated support and their disappointment in it. Disappointment with the received help or lack of information seems to also explain the appearance of PTSD symptoms. This interpretation follows Chan and Huak (27) research, where they indicated that support from superiors and colleagues was a significant negative predictor of psychiatric symptoms, including PTSD: when these factors were not provided, PTSD symptoms seemed more likely to emerge. On that account, lack of administrated help and knowledge may have contributed to PTSD symptoms, especially among those quarantined, who had no choice but to rely on health-care administration.

The present research has limitations that need to be addressed. Representativeness of the studied sample might be limited due to the lack of records concerning the number of those initially approached and reasons for some of them to refuse participation. This limited representativeness concerns both the general sample and the quarantine (+) and quarantine (-) groups. Moreover, the quarantine (+) group must have been extended due to under representativeness of quarantined individuals, which stands for the biggest limitation of the presented study. For that reason, the results cannot be related to only narrowed group of individuals who experienced quarantine with emotions and problematic issues connected with it. This makes the result of the study more exposed to the influence of some independent factors that should be researched and further addressed. Also, even though two questionnaires were used to assess psychopathological symptoms, no specific diagnosis could have been measured. Additionally, this study would profit from using more thorough questions concerning coping strategies, for instance, turning to religion. This would provide a light on the effect of this specific coping strategy, which in the presented study contradicts results previously obtained on similar matter. Finally, our study was not based on a longitudinal design, and thus causal associations and temporal patterns of changes in psychopathological manifestation cannot be concluded.

In summary, our research is a response to a current high demand for information on mental health among populations affected by the pandemic. It provides information about mental health, coping strategies, and their correlations in the general population, but most importantly regarding those quarantined, so individuals who were isolating and awaiting their test result or the COVID-19 symptoms to occur after contacting someone infected or individuals who has been worrying about their loved ones going through the same procedure. This information should be researched and adapted by policymakers and mental-health professionals. We have provided evidence that those quarantined are more likely to develop psychopathological symptoms, such as somatic symptoms, anxiety, insomnia, and PTSD symptoms of intrusion and hyperarousal. They are also more likely to turn to self-distraction as a coping strategy and for that reason specific policies concerning relevance of psychosocial interventions targeted at early psychopathological symptoms, regular access to medical information, and health monitoring should be revised and implemented. Furthermore, we found correlations between manifested coping strategies and psychopathology among the general population experiencing pandemic. Apart from that, presented research constitutes grounds for follow-up research as well as for creating personalized interventions aimed at improving or restoring control over psychological well-being among quarantined and not quarantined individuals during the time of a pandemic.

## Data Availability Statement

The raw data supporting the conclusions of this article will be made available by the authors, without undue reservation.

## Ethics Statement

The studies involving human participants were reviewed and approved by Ethics committee of the Wroclaw Medical University. The patients/participants provided their written informed consent to participate in this study.

## Author Contributions

JM, MC, DS, BM, TW, KF-W, DŁ, AK, and JR contributed to research conceptualization. JM, MC, DS, BM, TW, and KF-W managed data curation and formal analysis were performed by JM, MC, DS, BM, DŁ, and AK. JM, MC, DS, BM, and JR were responsible for funding acquisition, investigation, and methodology were performed by JM, MC, DS, BM, TW, KF-W, DŁ, JR, JM, and MC were project administrators, resources, and software were the responsibility of JM, MC, DS, TW, KF-W, AK, JR, BM, and DŁ. MB and JR provided supervision. All authors listed have made a substantial, direct and intellectual contribution to the work, and approved it for publication.

## Conflict of Interest

The authors declare that the research was conducted in the absence of any commercial or financial relationships that could be construed as a potential conflict of interest.

## Footnotes

^1^https://www.hhs.gov/answers/public-health-and-safety/what-is-the-difference-between-isolation-and-quarantine/index.html, accessed 15.12.2020 14:54.
